# Metformin attenuated sepsis-associated liver injury and inflammatory response in aged mice

**DOI:** 10.1080/21655979.2022.2036305

**Published:** 2022-02-13

**Authors:** Heng Song, Xiaojuan Zhang, Ruiqing Zhai, Huoyan Liang, Gaofei Song, Yangyang Yuan, Yanan Xu, Yan Yan, Lingxiao Qiu, Tongwen Sun

**Affiliations:** a General ICU, The First Affiliated Hospital of Zhengzhou University, Henan Key Laboratory of Critical Care Medicine, Zhengzhou Key Laboratory of Sepsis, Henan Engineering Research Center for Critical Care Medicine, Zhengzhou, China; b Academy of Medical Sciences, Zhengzhou University, Zhengzhou, China; c College of Bioinformatics Science and Technology, Harbin Medical University, Harbin, China; d Department of Respiratory Medicine, The First Affiliated Hospital of Zhengzhou University, Zhengzhou, China

**Keywords:** Metformin, lipopolysaccharide, sepsis-associated liver injury, aged mice, inflammatory response

## Abstract

Sepsis-associated liver injury is with poor survival in intensive care units. Metformin is well known for its therapeutic effects; however, its impact on treating liver injury due to sepsis remains poorly understood. This study investigated the therapeutic effects of metformin on aged mice suffering from sepsis-associated liver injury. Male C57BL/6 J mice aged (18–19 months) were divided into 3 groups: 1) intraperitoneal injection of sterile normal saline (C group), 12.5 mg/kg lipopolysaccharide (LPS) to induce sepsis-associated liver injury (LPS group), and 25 mg/kg metformin (MET) at 1 h after LPS injection (MET group). After 24 h, blood samples and liver tissue were collected for biochemical analysis. Histological assays revealed significantly elevated inflammatory infiltration and apoptosis in the liver, while metformin was found to relieve these aberrant features. The percentage of apoptotic cells decreased after metformin treatment (*P* < 0.05). Additionally, MET group had significantly reduced plasma alanine aminotransferase (ALT) and aspartate aminotransferase (AST) levels compared to the LPS group (*P* < 0.05). Furthermore, in the MET group, the mRNA levels of chemokines and inflammatory factors, TNF-α, IL-6, caspase-1, decreased markedly (*P* < 0.05). Metformin notably reversed the decreased phosphorylated AMP-activated protein kinase (p-AMPK) and PGC-1α expressions in the liver of septic rats. Metformin also inhibited PDK1, HIF-1α expression, including downstream inflammatory mediators, HMGB1 and TNF-α. Metformin attenuated inflammation and liver injury in septic aged mice. Most importantly, we report the effect of metformin on liver injury via the AMPK–PGC1α axis in septic aged mice for the first time.

## Graphical abstract

**Figure:**
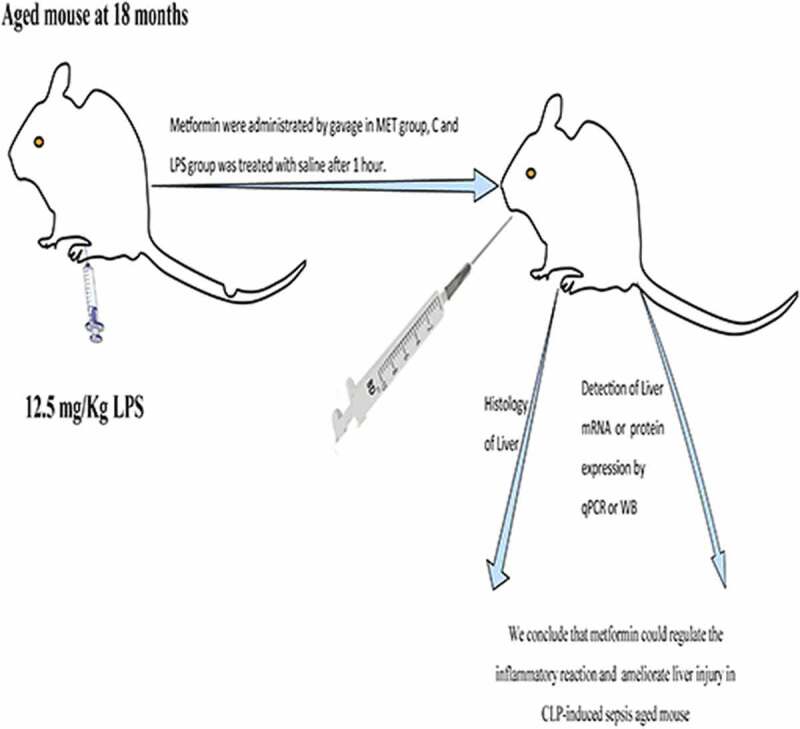

## Introduction

Sepsis is a life-threatening organ dysfunction caused by the dysregulated host response to infection [1], frequently resulting in disability and death [2]. It affects one-third of patients admitted to intensive care units (ICU), with an estimated mortality rate of 25–35% [3]. Strikingly, the incidence of sepsis in the elderly is relatively high. More than half of sepsis patients are 65 years and older [4–6]. Additionally, sepsis in older patients has a worse outcome, with higher rates of mortality, disability, long-term hospitalization, and severe organ dysfunction [7–9]. The pathogenesis and vital organ injury (especially the liver injury) due to sepsis leading to death are not fully understood. Moreover, there are hardly any effective sepsis-treatment strategies [10–12]. Therefore, it is urgent to explore new effective therapies to attenuate sepsis-induced inflammation and liver injury.

Metformin has long been a first-line drug treating type 2 diabetes, and in recent decades it was found to have a wide range of non-hypoglycemic effects, including anti-tumor, anti-inflammatory, anti-aging, and anti-microbial effects [13–16]. Potential mechanisms for metformin to exert these effects include increased AMPK activity and anti-reactive oxygen species effects, thereby reducing chronic inflammation and oxidative damage accumulation [17]. Metformin can also prevent the onset and progression of sepsis by inhibiting nuclear factor kappa-light-chain-enhancer of activated B cells (NF-κB) directly, independent of the AMPK-pathway activation [18], and thus exert an anti-inflammatory effect [19]. Additionally, metformin can protect lung tissue from oxidative injury induced by sepsis [20]. Our previous review also showed that before-admission metformin use was associated with lower mortality in adult septic patients with diabetes mellitus [21]. However, the role of metformin on inflammation during sepsis has not been studied in aged mice. This study is the first to investigate the effects of metformin on sepsis-induced liver injury in aged mice and decipher the possible mechanisms, which may provide a potential therapy for sepsis. In the present study we hypothesized that metformin may alleviate liver injury in aged septic mice by activating AMPK and thus upregulating PGC1α. We investigated the therapeutic effects of metformin on aged mice suffering from sepsis-associated liver injury.

## Materials and methods

### Construction of sepsis model

Thirty adult male C57BL/6 J mice (each weighing 30–40 g, 18 months old) were purchased from Charles River (Beijing, China). The experimental mice were housed in standard cages at a constant ambient temperature and humidity (22 ± 2°C, 40–60%, *n* = 5 per cage) and kept under a twelve-hour light/dark cycle with free food and drinking. Experiments were conducted after 1 week of acclimatization of the mice to the environment. The experimental animals were randomly divided into three groups (*n* = 10/group): physiological saline was injected intraperitoneally as control (C group); the animal model of sepsis was constructed by intraperitoneal injection of lipopolysaccharide (LPS) dissolved in physiological saline at 12.5 mg/kg (LPS group); and metformin (MetChemExpress; Monmouth Junction, NJ, USA) dissolved in physiological saline was injected intraperitoneally at 25 mg/kg at 1 h after completion of the LPS injection (MET group). The animal body temperature was recorded before and 18 h after LPS injection with an electronic rectal thermometer. This study was conducted in compliance with the National Institutes of Health (NIH) Guide for the Care and Use of Laboratory Animals, and the Ethical Review Committee of Zhengzhou University approved the experiments.

### Liver tissue and plasma sample collection

The mice were anesthetized using 0.6% pentobarbital sodium (40 mg/kg of body weight, IP) at 24 hours after LPS injection. The blood was collected with an anticoagulant tube containing ethylenediaminetetraacetic acid (EDTA) from retro-orbital sinus and centrifuged (2,500 rpm, 10 min, room temperature) to collect plasma, which was then stored at – 80°C for subsequent assays. The thorax of the mouse was opened to expose the thoracic cavity and cut the postcava, followed by the perfusion needle insertion into the apical region. A volume (approximately 100 mL) of pre-cooled saline was rapidly pushed into the left ventricle and aorta. Liver tissues were removed and stored at – 80°C and fixed in 4% paraformaldehyde for subsequent experiments.

### Biochemical analysis

Plasma alanine aminotransferase (ALT) and aspartate aminotransferase (AST) were measured using biochemical kits per manufacturer’s instructions (Nanjing Jiancheng Bioengineering Institute, China).

### Enzyme-linked immunosorbent assay (ELISA) for cytokines

Mouse ELISA Kits (CSB-E04741m; CSB-E04639m, CUSABIO, Wuhan, China) were used to determine plasma tumor necrosis factor α (TNF-α) and interleukin 6 (IL-6) levels. Briefly, the optical density (OD) of the plasma sample was measured at 450 nm to quantify values of TNF-α and IL-6 by comparing relative to each standard curve respectively.

### Histological and apoptosis analysis of mice liver

Hematoxylin and eosin (H&E) staining was performed to observe the degree of inflammatory cell infiltration. The liver tissue was embedded in paraffin and sliced into 3–5 μm sections. The H&E stained sections were observed under a light microscope. We quantified edema and hemorrhage within the liver tissue. To assess the liver lesions better, we chose three sections and three regions within each section to obtain the mean score as the final score for each animal. Terminal deoxynucleotidyl transferase-mediated deoxyuridine triphosphate-biotin nick end-labeling (TUNEL) staining assay (KeyGen, Nanjing, China) was used to label the fragmented DNA of apoptotic cells (brown nuclear staining) according to the manufacturer’s instructions.

### Western blot assay

The proteins from each liver tissue sample were extracted using a high radioimmunoprecipitation assay (RIPA) buffer (Solarbio Science & Technology Co., Ltd.,), and a bicinchoninic acid (BCA) protein assay kit (Solarbio Science & Technology Co., Ltd.,) was used to determine the concentrations. A small amount of the total protein (30 μg) per sample was subjected to sodium dodecyl sulfate–polyacrylamide gel electrophoresis (SDS-PAGE) and electrophoretically transferred onto a polyvinylidene fluoride (PVDF) membrane (Immobilon®-P, USA). The membrane was incubated with 5% (w/v) skim milk in the *tris*-buffered saline with Tween-20 (1‰) for 90 min at room temperature, and incubated overnight at 4°C with different primary antibodies (anti-AMPK (CST, Boston, USA; 1:1000), anti-p-AMPK, anti-PGC-1α, anti-PDK1, anti-AKT, anti-p-AKT, anti-MAPK p38, anti-p-p38, and anti-HIF-1α from CST, Boston, USA (1:1000 and anti-HMGB1anti-TNF-α, anti-GAPDH from Proteintech, Wuhan, China (1:5000)). The membrane was then washed three times and incubated with goat anti-rabbit IgG-HRP secondary antibody (Cwbio, Beijing, China 1:5000) for 90 min at room temperature. Glyceraldehyde 3-phosphate dehydrogenase (GAPDH) was used as an internal reference protein for total protein blotting. The process of Western blot has been described previously [22]. The Image J software was used to quantify the densitometry of bands.

### Reverse transcription-polymerase chain reaction analysis

The experimental process according to describe earlier [23]. TRIzol reagent (Takara, Tokyo, Japan) was used to extract total RNA from the liver. The concentration and purity of RNA were quantified by ultraviolet spectroscopy. The TaqMan Reverse Transcription Kit (UE, Suzhou, China) and a Gene Amp polymerase chain reaction (PCR) system were used for generating cDNA. Polymerase chain reaction using qPCR SYBR Green Master Mix (Yeasen, Shanghai, China). All the amplifications were repeated three times. Oligonucleotide primer sequences were: Caspase-1 forward primer: CTGAGGGCAAAGAGGAAGCA, reverse primer: AACTTGAGCTCCAACCCTCG. Hypoxia-inducible factor-1α (HIF-1α) forward primer: CAGCCAGCAAGTCCTTCTGA, reverse primer: TGCCTTAGCAGTGGTCGTTT. Interleukin 6 (IL-6) forward primer: TGATGCACTTGCAGAAAACA, reverse primer: ACCAGAGGAAATTTTCAATAGGC. Pyruvate dehydrogenase kinase-1 (PDK1) forward primer: GGATCCTGTCACCAGCCAAA, reverse primer: AGGCGTGATATGGGCAATCC. Peroxisome proliferative activated receptor, gamma, coactivator 1 alpha (PGC-1α) forward primer: CAGGAACAGCAGCAGAGACA, reverse primer: TGGAAGAACAGATGTGCCCC. Tumor necrosis factor-α (TNF-α) forward primer: CCACCACGCTCTTCTGTCTAC, reverse primer: AGGGTCTGGGCCATAGAACT. C–C motif chemokine ligand 3 (CCL3) forward primer: ACCATGACACTCTGCAACCA, reverse primer: GTGGAATCTTCCGGCTGTAG. C–C motif chemokine ligand 4 (CCL4) forward primer: CATGAAGCTCTGCGTGTCTG, reverse primer: GAAACAGCAGGAAGTGGGAG. C–C motif chemokine ligand 7 (CCL7) forward primer: CTGCTTTCAGCATCCAAGTG, reverse primer: TTCCTCTTGGGGATCTTTTG. C–C motif chemokine ligand 8 (CCL8) forward primer: TCTTTGCCTGCTGCTCATAG, reverse primer: GAAGGGGGATCTTCAGCTTT. β-actin (actin, beta) forward primer: AGTGTGACGTTGACATCCGT, reverse primer: GCAGCTCAGTAACAGTCCGC. β-actin was used as an endogenous control, and each sample was normalized according to its β-actin content. The gene expression data were set to 100% relative to the control group. The relative quantification of the target gene mRNA expression levels was calculated by the 2^−ΔΔCt^ method.

### Statistical evaluation

All data were presented as mean ± standard. The Student *t*-test (two-group comparisons) and one-way ANOVA (three-group comparisons) were used to determine differences. We performed Tukey’s post hoc test when there was a significant difference in ANOVA analysis. Differences were considered statistically significant at *P* < 0.05. The graphs were presented using GraphPad Prism 8.0.

## Results

In the present study we hypothesized that metformin may alleviate liver injury in aged septic mice via the AMPK–PGC1α axis. After performing the aged septic mice model, blood samples and liver tissue were collected for biochemical analysis, histopathology, reverse transcription-polymerase chain reaction (RT–PCR), and Western blot assays. We investigated the therapeutic effects of metformin in aged septic mice.

### The effects of metformin on body temperature and liver dysfunction in septic-aged mice

**Figure 1.**
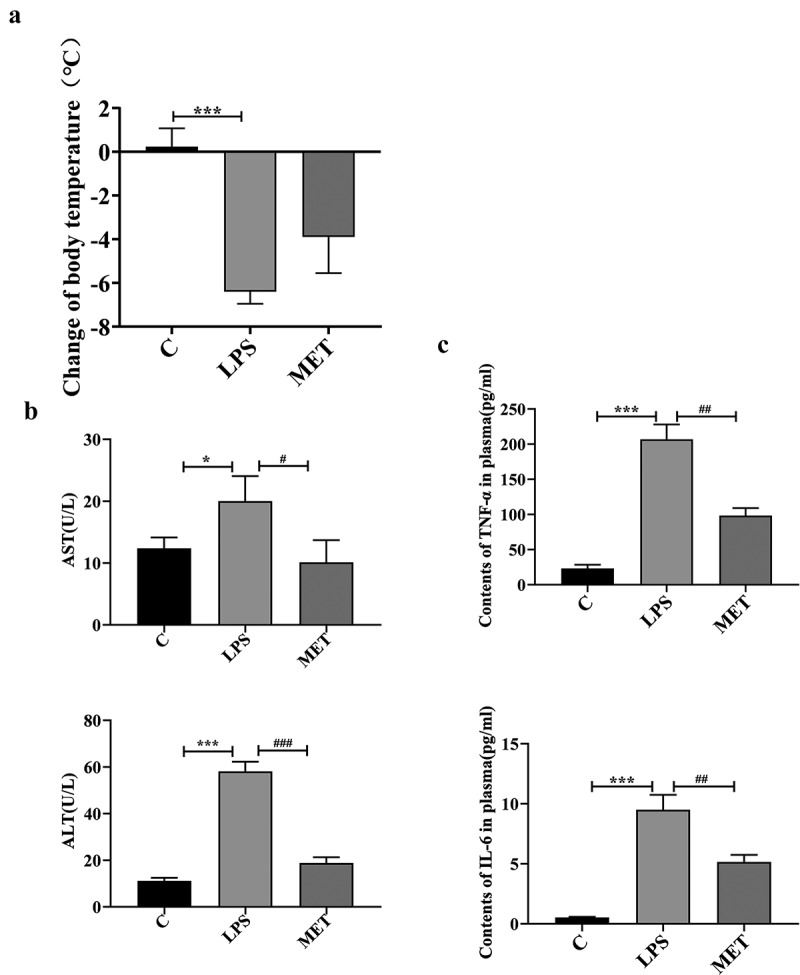
The effect of metformin on body temperature, plasma alanine aminotransferase (ALT) and aspartate aminotransferase (AST). (a) Changes in body temperature before and after 18 h of LPS injection among C group, LPS group, and MET group. (n = 5 per group) (b) Plasma ALT/AST levels before and 24 hours after the LPS injection in the three groups. (n = 3 per group) (c) Plasma TNF-α and IL-6 contents measured by ELISA. Values with *P < .05, **P < .01, and ***P < .001 are statistically significant between C group and LPS group, while values with #*P* < .05, ##*P* < .01, and ###*P* < .001 are statistically significant between LPS group and MET group.

The body temperature (BT) of mice was recorded before and 18 h after the LPS injection. The body temperature of LPS-administered mice was significantly lower than group C, with a mean decrease of 6.38°C (Figure 1(a), *P* < 0.05). However, the body temperature of metformin-treated mice decreased by a mean of 4.02°C. The data indicated no statistically significant differences in the BT changes between the LPS-administered and the metformin-treated mice. However, metformin had a certain reversal effect on hypothermia in the septic aged mice. In addition, the level of plasma ALT of the control mice increased significantly from 11.17 U/L to 58.11 U/L in the LPS-administered mice; whereas its level decreased to 18.86 U/L in the metformin-treated septic aged mice. Metformin also reduced the plasma AST levels (Figure 1(b), *P* < 0.05). Meanwhile, we quantified the contents of TNF-α and IL-6 in the plasma of three groups of mice. As expected, the contents of these two pro-inflammatory cytokines showed a significant decrease after metformin treatment (Figure 1(c), *P* < 0.05). The results demonstrated the beneficial effects of metformin in reversing sepsis-associated hypothermia and liver dysfunction in septic aged mice.

### Metformin ameliorated the pathological liver damage and cell apoptosis in septic aged mice

**Figure 2.**
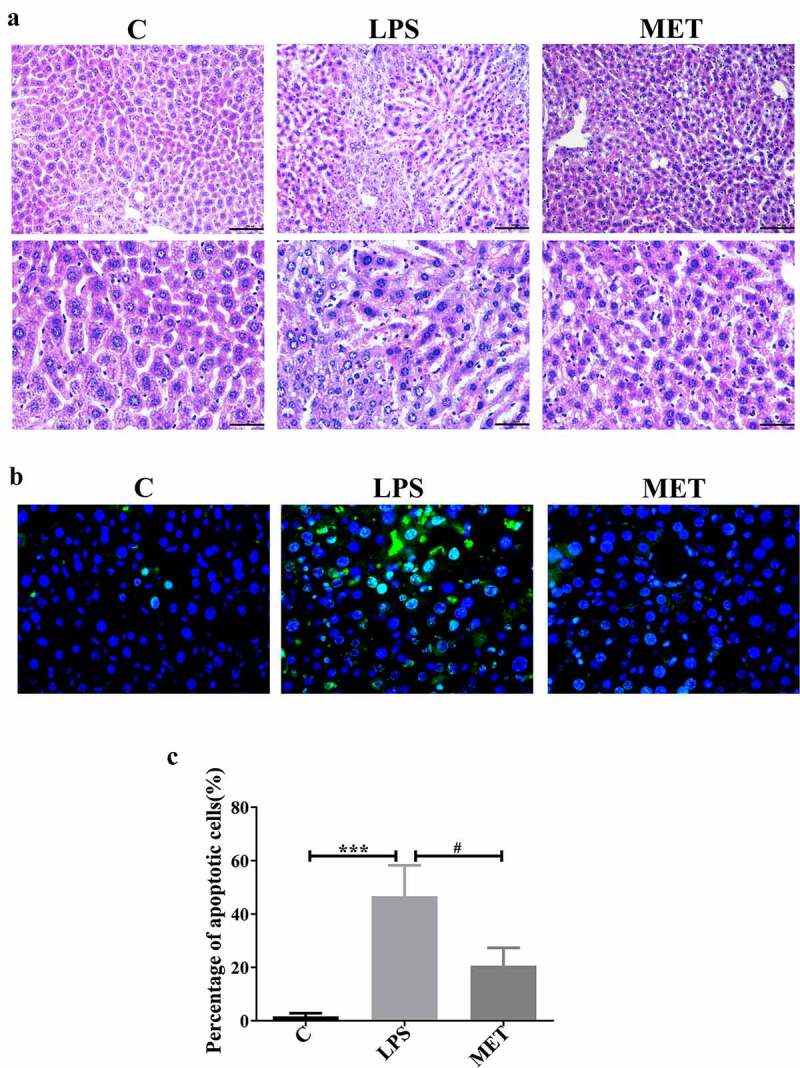
Effects of metformin on liver injury and inflammation in septic, aged mice. (a) H&E staining indicated the presence of inflammatory infiltrates, edema, and hemorrhage in the LPS group; however, metformin treatment ameliorated these abnormalities (scale bars = 100 μm/50 μm). (b) The TUNEL assay results in the three groups (scale bars = 50 μm). (c) The percentage of apoptotic cells in the three groups. (n = 3 per group).

We performed the H&E staining of the liver tissue to evaluate the organ pathological injury and hepatocyte apoptosis caused by LPS-induced sepsis. The LPS administration group showed significant destruction of hepatic lobular structures, edema, inflammatory cell infiltration, and hepatic hemorrhage compared to the control group, and these observations were mitigated in the metformin-treated mice (Figure 2(a)). We further conducted the TUNEL assay to confirm the role of metformin in hepatocytes apoptosis. The results showed that the number of apoptotic cells in the liver of mice in the LPS group was significantly increased compared with the C group; however, they were distinctly decreased in the MET group (Figure 2(b)). The percentage of apoptotic cells in the LPS group was markedly higher than the C group, and metformin administration substantially alleviated cell apoptosis (Figure 2(c), *P* < 0.05). These results were consistent with the results of H&E staining. Thus, metformin ameliorated liver pathological damage and cell apoptosis in septic aged mice.

### Metformin attenuated liver injury and inflammatory response in septic aged mice

**Figure 3.**
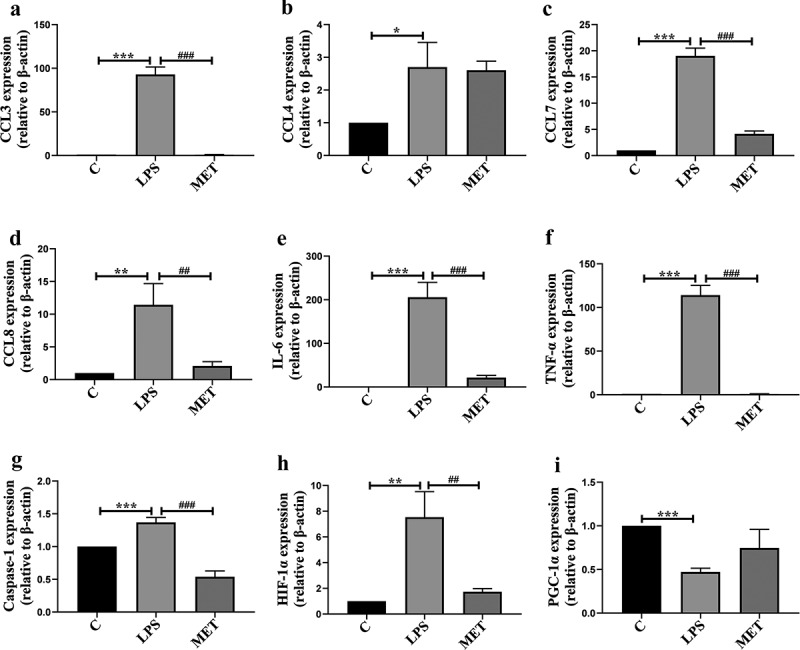
(a–h) The mRNA expression levels of chemokines and inflammatory genes among C group, LPS group, and MET group. (n = 3 per group) (i) The mRNA expression levels of PGC-1α in the three groups. (n = 3 per group).

Overwhelming inflammatory response derived from a cytokine storm is the main cause of organ dysfunction and lethality in sepsis [24]. We analyzed the expressions of relevant cytokines and chemokines in septic aged mice. We found LPS-related sepsis visibly upregulated the mRNA expression of IL-6, TNF-α, pyrocytosis-related factors (such as caspase-1), hypoxia-inducible factors (such as HIF1-α), and chemokines. In contrast, metformin administration markedly reduced the mRNA levels of these cytokines and chemokines compared with the LPS group (Figure 3(a-h), *P* < 0.05). It was also observed that the mRNA level of PGC1-α decreased in the LPS-induced septic aged mice compared to the C group, whereas metformin reversed the LPS-induced decreased expression of PGC1-α (Figure 3(i), *P* < 0.05).

**Figure 4.**
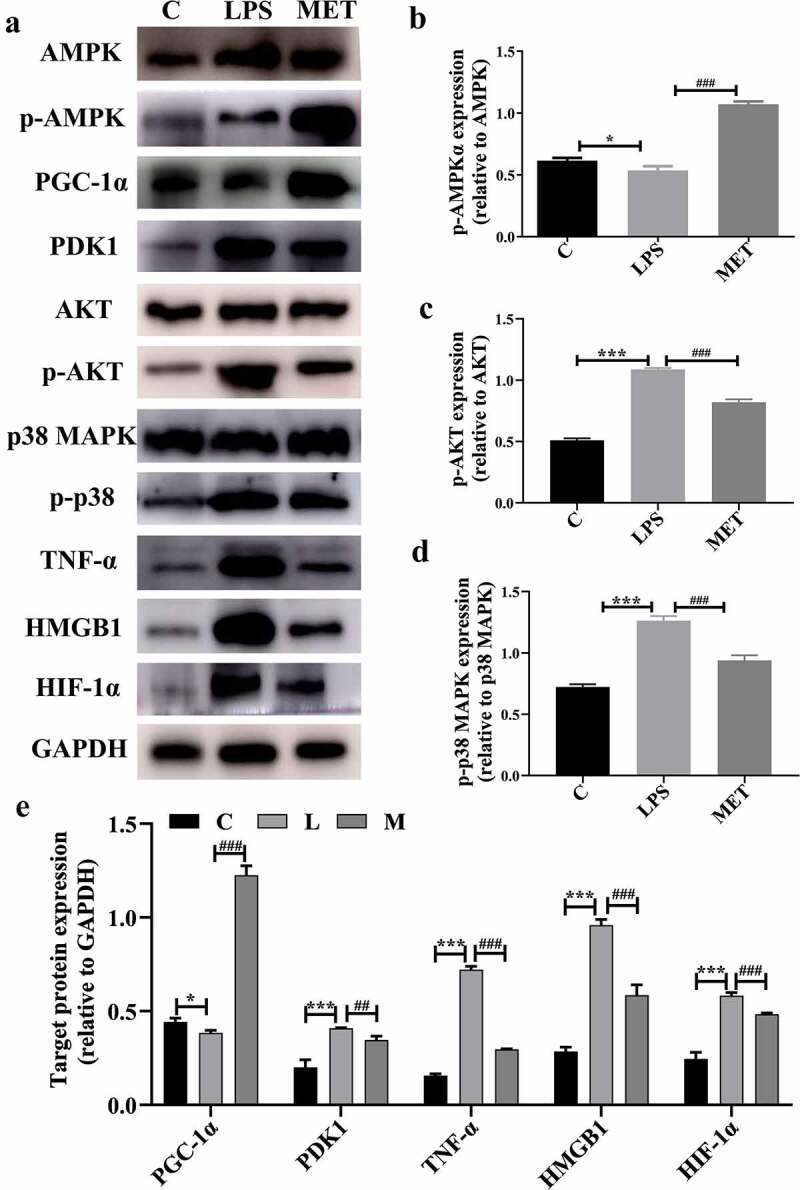
(a) Protein expression levels among C group, LPS group, and MET group. (b–d) The level of protein expression in the activated AMPK/Akt/p38 signaling pathway among the three groups. (e) The expression of target protein in the three groups. (n = 3 per group).

Studies have shown that suppression of PGC1-α reprogrammed the metabolic energy and altered mitochondrial morphology, while reactivation of PGC-1 reduced oxidative stress and improved organ function [25,26]. Next, we measured changes in relevant target proteins, such as PGC1-α, PDK1, HIF-1α, and related inflammatory factors, to understand the potential mechanisms by which metformin attenuated inflammatory response and liver injury in aged septic mice. We observed that LPS administration activated the AKT signaling pathway proteins such as MAPK p38, TNF-α, and HMGB1. As expected, metformin attenuated inflammation and liver injury by activating the AMPK phosphorylation that inhibited the AKT signal activation. Moreover, metformin reversed the PGC1-α decrease and the PDK1 and HIF-1α increase caused by LPS (Figure 4, *P* < 0.05). These results clearly indicated that metformin attenuates liver injury and inflammatory responses, possibly by activating the AMPK-PGC1-α axis in aged septic mice.

## Discussion

This study suggested that metformin may mitigate inflammatory response and liver injury via the AMPK–PGC1-α axis in septic aged mice. Our findings imply metformin as a potential therapeutic option for moderating inflammatory response on sepsis-associated liver injury in aged mice.

Most animal studies on sepsis employed young animals. Previous studies have reported an increased incidence of sepsis and mortality with age due to increased plasma inflammatory cytokine levels and local inflammatory responses in aged mice [27–29]. Studies on adult animals have restricted our knowledge and understanding of treatment strategies for elderly patients with sepsis. Therefore, we used aged mice in this study to establish a sepsis model and to evaluate the effect of metformin on sepsis-related liver injury.

A study reported that metformin alleviated liver dysfunction by decreasing hepatic lipid accumulation and serum levels of ALT/AST in ob/ob mice [30]. As known, the ALT/AST activities are appropriate tests for estimating the extent of liver injury. Similarly, our results revealed that metformin treatment, compared to LPS administration, markedly decreased the ALT/AST levels in circulation, suggesting a favorable effect of metformin in protecting the liver.

Recently, a study reported that elevated BT of patients with severe sepsis or septic shock on ICU admission predicted increased survival [31]. Yet another study reported that mortality in non-elderly patients with sepsis decreased with fever and increased with hypothermia, whereas no correlation between mortality and BT was found in elderly patients [32]. Our study found that metformin administration could reverse the hypothermia caused by LPS in aged mice, although there was no statistical difference. The interaction of BT, age, and other confounding factors on the prognosis of sepsis had no noticeable results. The metformin mechanism on BT of septic mice needs to be further explored.

Our research is consistent with the previous report that metformin protects against sepsis in rats [20], which may be attributed to its anti-inflammatory effects and reduction in neutrophil accumulation. We found that metformin activated the AMPK signaling pathway and reversed the decrease in PGC1-α expression and the increase in PDK1 expression in the liver of aged septic mice. Additionally, the related inflammatory factors, such as TNF-α, HMGB1, were downregulated in the MET group. We also found that metformin reduced LPS-induced elevated expression of mRNA for pro-inflammatory factors, such as TNF-α, IL-6, caspase-1, and HIF1-α. These findings were in line with previous studies [33,34] that showed AMPK could limit the activation of downstream inflammatory targets in various situations. We observed that metformin reduced the increased mRNA levels of chemokines caused by LPS. Chemokines play a crucial role in recruiting inflammatory cells in sepsis that contribute to neutrophil infiltration leading to multiple organ dysfunction and failure [35]. However, the exact underlying mechanisms remain to be further explored.

When sepsis occurs, the immune response caused by the invading pathogen fails to restore homeostasis, eventually leading to a pathological syndrome characterized by persistent hyperinflammation and immunosuppression [36]. Cellular bioenergetics and metabolism play a key role in the regulation of immunity during acute inflammation. Inflammation in sepsis involves both adaptive and innate immunity [37], and the early immune response requires a high-energy metabolic state supported by glucose-dependent ATP production, mainly provided by glycolysis.

Metformin inducing the activation of the energy metabolism switch AMPK, has been well documented [38]. The AMPK could inhibit bioenergetic reprogramming (aerobic glycolysis) in macrophages and monocytes [39]. AMPK signaling pathway plays a substantial role in cell cycle, energy metabolism reprogramming, and autophagy [40–42]; during hypoxia and oxidative stress, AMPK is activated and promotes metabolic reprogramming.

The transcriptional coactivator, PGC-1α, mediates many biological processes related to energy metabolism, especially the control of mitochondrial biogenesis and oxidative metabolism [43,44]. The suppressed PGC-1α caused by LPS administration results in cardiac energy metabolic reprogramming and functional derangements [26]. Meanwhile, the increased PGC-1α expression could alleviate endotoxin-induced acute kidney injury, mitochondrial injury, and superoxide accumulation [45]. This study found that metformin reversed the LPS-induced downregulation of PGC-1α expression in aged mice. A previous study showed that cardiac AMPK mediates PGC-1α levels by post-myocardial infarction [46]. In addition, activating the AMPK–PGC‐1α signaling pathway could inhibit apoptosis and enhance energy metabolism [47]. Our results were in line with the previous findings.

PDK1 was reported as the downstream regulator of PGC1-α that improved inflammation and metabolism by inhibiting the PDK1 expression [48]. Furthermore, a previous study showed that the PDK activity blockage in mice represses macrophage M1 polarization, reducing adipose tissue inflammation and insulin resistance [49]. PDK1 enhanced glycolysis in macrophages co-incubated with LPS, thereby promoting M1 polarization of macrophages [50]. Several studies showed that glycolysis regulates activation of M1 macrophages and other immunocytes, such as dendritic cells [51–55].

It is well known that HIF-1α is the primary glycolysis-regulating molecule. Cells respond to hypoxia through HIF-1α mediated upregulation of glucose transport proteins and glycolysis-related enzymes, such as phosphofructose kinase and PDK1 [56], which interrupt pyruvate dehydrogenase enzyme and reduce the mitochondrial glucose oxidation in the tricarboxylic acid cycle.

Metformin inhibited inflammatory infiltration by reducing the recruitment of macrophages [57], and decreased expression of inflammatory factors, such as HMGB1, IL-1β, and IL-6 [58]. Earlier studies demonstrated that metformin reduced mortality in a mouse model of lethal endotoxemia by inhibiting HMGB1 release; inhibition of NF-κB-induced TNF-α activation and AMPK activation were the mechanisms contributing to the protective effects [59,60]. Our results suggest that AMPK upregulated the PGC1-α expression, which then inhibited PDK1 and HIF-1α expression.

There are some limitations in the present study. We only conducted in vivo experiments and lacked in vitro functional experiments. The immunomodulatory effect of metformin seems to have a potential role in anti-sepsis treatment, but the exact mechanism remains to be studied and elucidated in the future.

## Conclusion

Our findings showed that metformin attenuated liver injury and inflammatory response in LPS-induced septic aged mice. Mechanistically, metformin may exert a protective effect on liver injury in septic aged mice via the AMPK–PGC-1α axis. Our study may provide new insights on metformin-based strategy for sepsis treatment in the elderly, although the mechanism requires further research and confirmation.
